# NMR Relaxivities of Paramagnetic Lanthanide-Containing Polyoxometalates

**DOI:** 10.3390/molecules26247481

**Published:** 2021-12-10

**Authors:** Aiswarya Chalikunnath Venu, Rami Nasser Din, Thomas Rudszuck, Pierre Picchetti, Papri Chakraborty, Annie K. Powell, Steffen Krämer, Gisela Guthausen, Masooma Ibrahim

**Affiliations:** 1 Karlsruhe Institute of Technology (KIT), Institute of Nanotechnology (INT), Hermann-von-Helmholtz-Platz 1, 76344 Eggenstein-Leopoldshafen, Germany; aiswarya.venu@kit.edu (A.C.V.); pierre.picchetti@kit.edu (P.P.); papri.chakraborty@kit.edu (P.C.); 2 LNCMI-EMFL, CNRS, INSA-T and UPS, Université Grenoble Alpes, Boîte Postale 166, CEDEX 9, 38042 Grenoble, France; rami.nasser-din@lncmi.cnrs.fr; 3 Karlsruhe Institute of Technology (KIT), MVM-VM, Adenauerring 20b, 76131 Karlsruhe, Germany; thomas.rudszuck@kit.edu; 4 Institute of Inorganic Chemistry, Karlsruhe Institute of Technology (KIT), Engesserstrasse 15, 76131 Karlsruhe, Germany; 5 Institute for Quantum Materials and Technologies (IQMT), Karlsruhe Institute of Technology (KIT), Hermann-von-Helmholtz-Platz 1, 76344 Eggenstein-Leopoldshafen, Germany; 6 Karlsruhe Institute of Technology (KIT), EBI-WCWT, Adenauerring 20b, 76131 Karlsruhe, Germany

**Keywords:** polyoxometalates, nuclear magnetic resonance imaging, paramagnetic relaxation enhancement, lanthanides, relaxivity, dysprosium, erbium

## Abstract

The current trend for ultra-high-field magnetic resonance imaging (MRI) technologies opens up new routes in clinical diagnostic imaging as well as in material imaging applications. MRI selectivity is further improved by using contrast agents (CAs), which enhance the image contrast and improve specificity by the paramagnetic relaxation enhancement (PRE) mechanism. Generally, the efficacy of a CA at a given magnetic field is measured by its longitudinal and transverse relaxivities *r*_1_ and *r*_2_, i.e., the longitudinal and transverse relaxation rates *T*_1_^−1^ and *T*_2_^−1^ normalized to CA concentration. However, even though basic NMR sensitivity and resolution become better in stronger fields, *r*_1_ of classic CA generally decreases, which often causes a reduction of the image contrast. In this regard, there is a growing interest in the development of new contrast agents that would be suitable to work at higher magnetic fields. One of the strategies to increase imaging contrast at high magnetic field is to inspect other paramagnetic ions than the commonly used Gd(III)-based CAs. For lanthanides, the magnetic moment can be higher than that of the isotropic Gd(III) ion. In addition, the symmetry of electronic ground state influences the PRE properties of a compound apart from diverse correlation times. In this work, PRE of water ^1^H has been investigated over a wide range of magnetic fields for aqueous solutions of the lanthanide containing polyoxometalates [Dy^III^(H_2_O)_4_GeW_11_O_39_]^5–^ (**Dy-W_11_**), [Er^III^(H_2_O)_3_GeW_11_O_39_]^5–^ (**Er-W_11_**) and [{Er^III^(H_2_O)(CH_3_COO)(P_2_W_17_O_61_)}_2_]^16−^ (**Er_2_-W_34_**) over a wide range of frequencies from 20 MHz to 1.4 GHz. Their relaxivities *r*_1_ and *r*_2_ increase with increasing applied fields. These results indicate that the three chosen POM systems are potential candidates for contrast agents, especially at high magnetic fields.

## 1. Introduction

Nuclear magnetic resonance (NMR) studies involving paramagnetic systems have been the subject of research in fields such as biochemistry, medicine, and material science [1,2]. The presence of an unpaired electron originating from paramagnetic molecules significantly affects the NMR behavior of the entire system [3,4]. The paramagnetic effects, which arise due to the hyperfine interactions between a nuclear spin *I* and the unpaired electronic spin *S* of the paramagnetic center, can be broadly categorized into two types. First, the NMR chemical shift range can be largely expanded over a wide spectral range, for example ppm range (δ-para) up to 100 for ^1^H. Even more than 1000 ppm for heavier ligand atoms such as ^13^C or ^15^N can be observed due to the large magnetic moment of unpaired electrons [5,6]. Second, the presence of an unpaired electron causes a faster nuclear spin relaxation that leads to the enhancement of longitudinal, *R*_1_ = 1/*T*_1_, and transverse, *R*_2_ = 1/*T*_2_, nuclear spin relaxation rates. This effect is commonly called paramagnetic relaxation enhancement (PRE). In this context, Curie spin relaxation is an important contributor to the water relaxivity in complexes of certain lanthanide ions (e.g., Tb, Dy, Ho, Er) due to their high magnetic moments and short electronic relaxation times, which limit the efficiency of Solomon-Bloembergen-type relaxation. The alignment of paramagnetic ions increases with field and inverse temperature according to Curie’s law. Thus, in high fields, where the Curie spin becomes large, nuclear transverse relaxation may be dominated by interaction with the Curie spin [7].

Magnetic anisotropy is one of the most important properties of the paramagnetic ions for technological applications and plays a particularly important role in the development of single-molecule magnets (SMMs) [8]. The anisotropic distribution of electrons in the 4f orbitals of lanthanide ions can be prolate (axially elongated) (Pm^III^, Sm^III^, Er^III^, Tm^III^, and Yb^III^) or oblate (equatorially elongated) (Ce^III^, Pr^III^, Nd^III^, Tb^III^, Dy^III^, and Ho^III^) (see Table S1) [9]. As a result of the anisotropy in the ground state of paramagnetic ions, a Larmor frequency difference occurs between the Ln-coordinated and the bulk water molecules, which is proportional to the external magnetic field [10]. Moreover, the Ln anisotropies strongly influence both paramagnetic NMR effects of solutions containing Ln complexes, the shifts [11], and the relaxation properties of the neighboring nuclei [12].

Paramagnetic relaxation enhancement (PRE) is usually explored in the field of magnetic resonance imaging (MRI) and NMR spectroscopy for the development of contrast agents and the structural determination of biomolecules, respectively [13].

Traditional contrast agents are mainly based on a paramagnetic gadolinium metal ion due to its seven unpaired electrons, high magnetic moment, and long electron spin relaxation time (10^−9^ s). However, recent information has raised new concerns about their toxicity [14]. Alternatively, other paramagnetic lanthanides are promising candidates to be used as contrast agents and among them dysprosium, due to its asymmetric/anisotropic electronic ground state (^6^H_15/2_) and a very high magnetic moment (*μ*_eff_  = 10.6 μB) may improve relaxivity [3,15].

Another important factor concerning MRI technologies is the use of ultra-high magnetic field instruments to improve sensitivity and spatial resolution. This is due to the strong dependency of signal to noise ratio in relation to the applied magnetic field (*B*_0_) [16,17]. In the past few decades, a different range of magnetic fields has been utilized depending on the various field of applications. The currently used magnetic field for clinical MRI systems is at a maximum of 11.7 T [18]. The preclinical system and the small animal imaging instruments are operated with up to 21.1 T. The maximum magnetic field of 28.2 T is used in NMR for the structural determination of small organic molecules or large biomolecules. MRI technologies with ultra-high magnetic fields require the use of new materials as contrast agents [16]. Moreover, for characterization of CAs over the entire field range of interest, results from NMR spectrometers using different magnet types need to be combined. These include fast field cycling techniques, permanent magnets, superconducting magnets and resistive high field magnets [19].

Recently, we have investigated NMR relaxivity of heterometallic high-spin molecular clusters [Fe_10_Ln_10_(Me-tea)_10_(Me-teaH)_10_(NO_3_)_10_] (lanthanides (Ln) Ln = Gd^III^, Tb^III^, Dy^III^, Er^III^ and Tm^III^) {Fe_10_Ln_10_} [20,21] and [Ln_30_Co_8_Ge_12_W_108_O_408_(OH)_42_(OH_2_)_30_]^56−^ (Ln = Gd^III^, Dy^III^, Eu^III^, and Y^III^) {Ln_30_Co_8_} [22]. The relaxivities of {Fe_10_Ln_10_} and {Ln_30_Co_8_} clusters in water were measured over a wide range of ^1^H Larmor frequencies from 10 MHz up to 1.4 GHz. The alteration of the lanthanide ions (at structurally similar sites within the same scaffold) in {Fe_10_Ln_10_} and {Ln_30_Co_8_} made it possible to differentiate the relaxation impacts of electronic states and molecular dynamics. The transverse relaxivity was found to increase with the field, whereas field dependence of the longitudinal relaxivities of these molecules depends on the nature of the lanthanide.

Apart from the electronic properties of the paramagnetic centers, the relaxivities depend on several other structural and dynamic features such as the number of bound water molecules, their rate of exchange with the bulk, size, clustering and aggregation, tumbling, diffusion and rotational correlation times [2,23].

**Figure 1:**
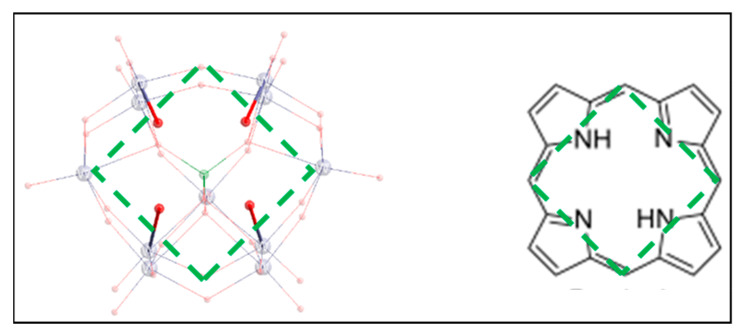
Analogy of monolacunary POM ligand [GeW_11_O_39_]^8−^ with porphyrin.

MRI contrast agents increase both *r*_1_ and *r*_2_ to different extents depending on their nature as well as on the applied magnetic field [24,25]. Accordingly, the development of new contrast agents is also a demanding area of research, which aims to increase their efficiency and sensitivity with increasing field. Field-dependent measurements of relaxivity are thus important to characterize new potential contrast agents. In this quest, one of the approaches we took was to synthesize and analyze the NMR relaxometry of ultrahigh-spin polyoxometalates (POM)-based heterometallic clusters in aqueous solutions [22]. The usage of Ln-POM nanocomposites as host/guest assemblies for contrast agents has also been reported by other groups [26,27,28]. POMs, as anionic metal oxide clusters, bear many properties that make them attractive for applications in various fields such as catalysis, magnetism, imaging, optics, medicine and also have interesting electrochemical properties [29,30,31,32,33,34]. Lacunary POMs or POM ligands are usually synthesized from parent plenary precursors by removal of one or more MO_6_ octahedra. Various types and the number of spin-coupled paramagnetic centers (d- or f-block), usually bridged via μ_2_-oxo/hydroxo can be incorporated into the structurally well-defined vacant sites of the lacunary POMs. This leads to the formation of monomeric dimeric, trimeric and tetrameric assemblies [35,36]. POM ligands can be viewed as an inorganic analogue of the porphyrins (Figure 1). By analogy, a paramagnetic metal containing polyoxometalates (PM-POMs) could be designed and investigated as contrast agents. As part of our continuous research in this field, we report another approach to investigate PRE of POM molecules that contain homometallic paramagnetic lanthanides. Thus, NMR relaxometry of aqueous solutions containing POMs K_5_[Dy(H_2_O)_4_GeW_11_O_39_]·16H_2_O (**Dy-W_11_**), K_5_[Er(H_2_O)_3_GeW_11_O_39_]·20H_2_O (**Er-W_11_**) [37], and (NH_2_Me_2_)_13_Na_3_[{Er(H_2_O)(CH_3_COO)(P_2_W_17_O_61_)}_2_]·40H_2_O (**Er_2_-W_34_**) [38] has been carried out at ^1^H Larmor frequencies from 20 MHz to 1.4 GHz.

### Structure of the POMs

**Figure 2:**
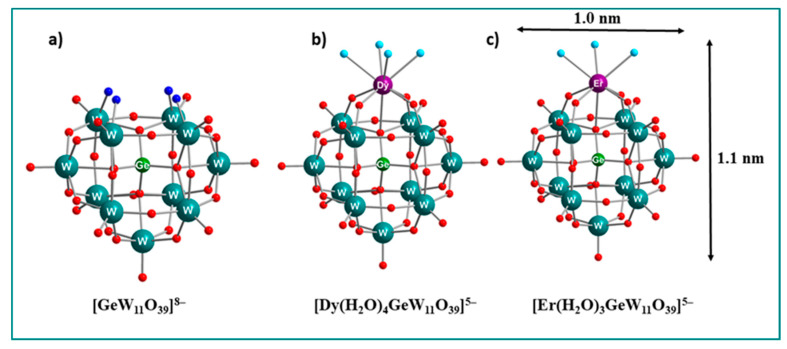
Ball-and-stick representation: (**a**) Keggin POM ligand [GeW_11_O_39_]^8−^. (**b**) [Dy(H_2_O)_4_GeW_11_O_39_]^5−^. (**c**) [Er(H_2_O)_3_GeW_11_O_39_]^5−^. Color scheme: O, red; aqua ligand, turquoise; four oxo ligands that coordinate to paramagnetic metal ions, dark blue.

Monolanthanide-substituted Keggin-type POMs: The Keggin POMs [Dy^III^(H_2_O)_4_GeW_11_O_39_]^5−^ (**Dy-W_11_**) and [Er^III^(H_2_O)_3_GeW_11_O_39_]^5−^ (**Er-W_11_**) contain one monolacunary Keggin [α-GeW_11_O_39_]^8−^ subunit and one lanthanide metal ion. This occupies the position that has been formed by loss of a W–O_t_ group from the plenary [α-GeW_12_O_40_]^4−^ anion, which consists of a central {GeO_4_} tetrahedron surrounded by four vertex-sharing {W_3_O_13_} triads (Figure 2). Mass spectrometric studies were carried out to check the stability of the compounds in solution which indicated that the Ln metal ion remains attached to the POM skeleton. However, the closed three-dimensional (3D) framework architecture, that was built by connecting POM moieties via K ion linkers, collapses in solution. The two isostructural POMs are different only in their number of exchangeable aqua ligands that are coordinated to Ln ions. There are three terminal water ligands in **Er-W_11_** due to the smaller ionic radius of Er^III^ ion compared to Dy^III^ ion which has four terminal water ligands [37].

**Figure 3:**
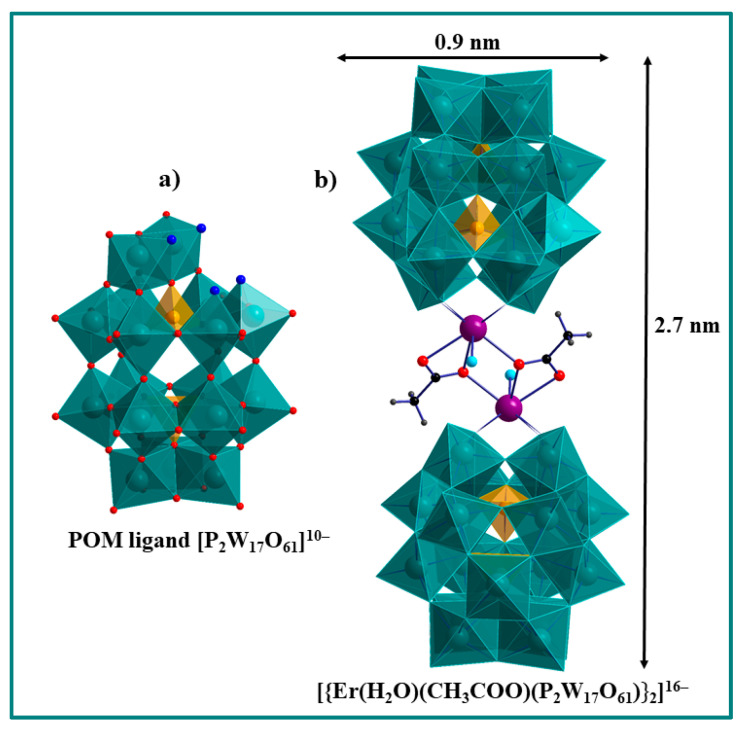
Combined polyhedral/ball-and-stick representation: (**a**) Wells-Dawson POM ligand [P_2_W_17_O_61_]^10−^. (**b**) [{Er(H_2_O)(CH_3_COO)(P_2_W_17_O_61_)}_2_]^16−^. Color scheme: O, red; aqua ligand, turquoise; four oxo ligands that coordinate to Er metal ions, dark blue; WO_6_ octahedra, teal; PO_4_ tetrahedra, yellow; Er, violet; C, black; H, grey.

Er-substituted Wells-Dawson-type POM: The anionic unit of [{Er^III^(H_2_O)(CH_3_COO)(P_2_W_17_O_61_)}_2_]^16−^ (**Er_2_-W_34_**) consists of dinuclear erbium(III) core, [{Er(H_2_O)(CH_3_COO)}_2_]^4+^, which is sandwiched between two monolacunary Wells-Dawson [P_2_W_17_O_61_]^10−^ units. Each Er^III^ in these units takes up the void (monolacunary) site of [P_2_W_17_O_61_]^10−^ and is coordinated to the four available oxygen atoms. The two {ErP_2_W_17_O_61_} units are bridged by acetate groups in the η^1^:η^2^:μ_2_ fashion. Each Er(III) ion is eight coordinate with square antiprismatic geometry. Thus, every Er^III^ ion in these units is coordinated by four O atoms of the POM ligand, two oxygen atoms of an acetate ligand, one O atom of the other acetate ligand and one terminal aqua ligand (Figure 3). The mass spectrometry data suggest that the polyanion **Er_2_-W_34_** is fragmented into monomeric species [38]. In some cases, MS performed under vacuum and μM conditions does not necessarily detect the species in solution, but rather captures the fragments of complexes that exist in the gas phase. Furthermore, the detection probability of the parent molecule during the transition from solution to gas phase decreases due to the weaker hydrophobic and stronger electrostatic interactions among gas phase assemblies compared to the solution phase [39]. Magnetic properties of the isolated Ln(III) are summarized in Table 1.

## 2. Results and Discussions

### 2.1. Water ^1^H Relaxation Measurements

To investigate the paramagnetic relaxation enhancement of the POMs [Dy^III^(H_2_O)_4_GeW_11_O_39_]^5–^ (**Dy-W_11_**), [Er^III^(H_2_O)_3_GeW_11_O_39_]^5−^ (**Er-W_11_**) and [{Er^III^(H_2_O)(CH_3_COO)(P_2_W_17_O_61_)}_2_]^16−^ (**Er_2_-W_34_**), longitudinal and transverse relaxivity measurements were performed at NMR Larmor frequencies from 20 MHz to 1.4 GHz. The detailed experimental procedures are explained in the previous publications [20,21,22,40]. For the current studies, pure crystals of POMs were dissolved in 9:1 D_2_O/H_2_O, and sets of five dilutions were prepared: 0-, 0.2-, 0.4-, 0.6-, 0.8- and 1-mM of complex. The investigated clusters are soluble and form aqueous solutions which remain clear after the NMR measurements have been performed (Figure S2). The dependence of *T*_1_ and *T*_2_ on POM dilutions were measured with different NMR magnetic fields with ^1^H-NMR Larmor frequencies ranging from 20 MHz to 1.4 GHz. Due to the limited availability of high power (24 MW) resistive magnets (≥800 MHz), only 1-, 0.6-, 0.2- and 0-mM dilutions were measured with the resistive magnet (≥800 MHz). Inversion recovery, progressive saturation recovery and Carr-Purcell-Meiboom-Gill (CPMG) multi-echo pulse sequences were used to measure the *T*_1_ and *T*_2_, respectively. All compounds exhibit a monoexponential relaxation curve that remains unchanged, when the solution is stored for a long time (several weeks). This indicates that the solution of the clusters is stable and no phase separation occurs. Both longitudinal and transverse relaxation rates of these POM clusters were determined as a function of concentration and, at each magnetic field, a linear relation was observed, as expected for a homogenous solution.

### 2.2. Factors Influencing PRE

PRE properties of the studied POM metal clusters can be derived from the ^1^H relaxation of water molecules of POM clusters in solution. PRE correlates with the structure and electronic properties of the POM metal clusters, which act as water ^1^H relaxing agents. The water molecule in the vicinity of the paramagnetic metal center follows a different relaxation behavior which reduces both longitudinal and transverse relaxation time. PRE also depends on the dynamics. Inner and outer sphere contributions are commonly distinguished. For the former the hydration number *q* of the molecules is an important factor, which also determines the efficiency of a molecule as a potential contrast agent. The theoretical description is governed by many parameters, such as the effective magnetic moments of the paramagnetic metal ions, their electron spin relaxation times, *T*_i,e_ (i = 1; 2), the tumbling rate of the complex, **τ**_R_**^−^**^1^, the residence time of water molecules near the paramagnetic center, **τ**_M_, as well as diffusion processes that are relevant for outer sphere contributions. For transverse relaxivities and high magnetic fields, Curie mechanisms, i.e., interaction of the nuclear spin with the thermal average of the electron spin, need to be taken into account [41].

### 2.3. Stabilty Studies of Er_2_-W_34_ in Solution

**Figure 4:**
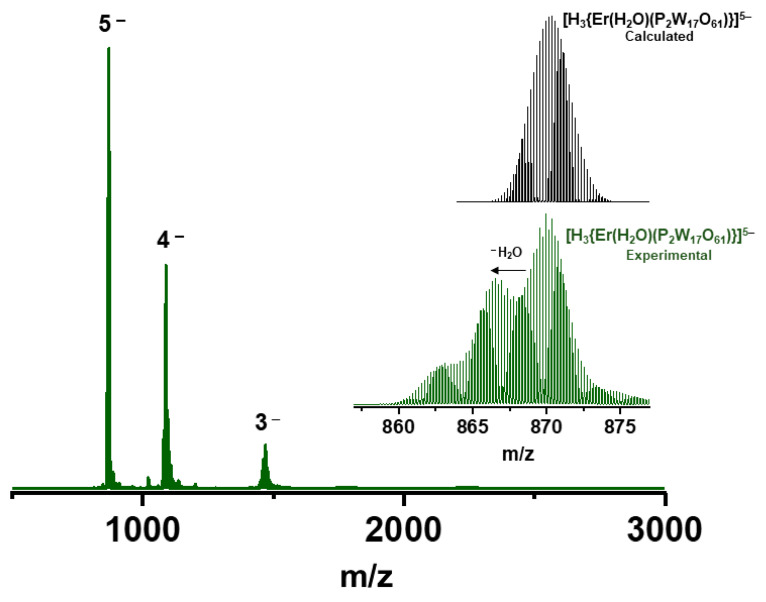
Negative ion electrospray ionization mass spectrometry (ESI MS) of **Er_2_-W_34_** sample having 0.2 mM concentration taken from the NMR tube and diluted further in 1:1 ACN/H_2_O. The 5− region is expanded in the inset and the peaks are compared with their calculated isotope pattern.

Electrospray ionization mass spectrometry (ESI-MS): Since our previously reported mass spectrometric (MS) studies on a dilute solution of **Er_2_-W_34_** have shown the fragmentation of the POM in gas phase, we decided to carry out ESI MS measurement of a diluted **Er_2_-W_34_** sample (0.2 mM taken from the NMR tube) in order to investigate their solution phase behavior (Figure 4). This was performed by further dilution in 1:1 ACN/H_2_O to μM concentration and produced spectra similar to our reported ones [39].

As discussed earlier, MS is a harsh technique of analysis. We therefore decided to perform a comparatively soft method of analysis.

**Figure 5:**
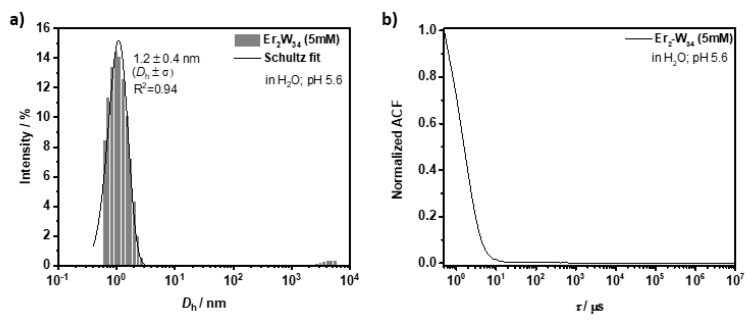
DLS analysis on **Er_2_-W_34_** (5 mM) at pH 5.6 in water. (**a**) Hydrodynamic diameter of **Er_2_-W_34_**. (**b**) Corresponding normalized autocorrelation function.

**Figure 6:**
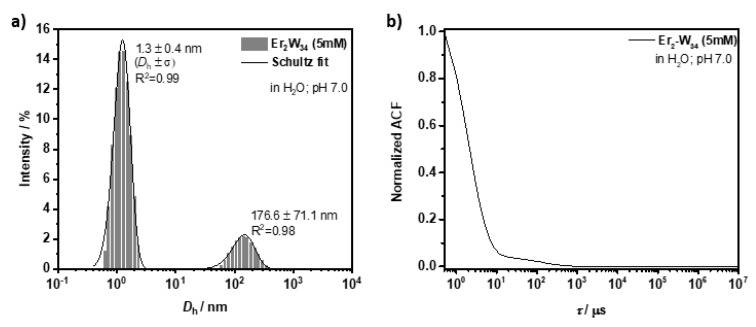
DLS analysis on **Er_2_-W_34_** (5 mM) at pH 7.0 in water. (**a**) Hydrodynamic diameter of **Er_2_-W_34_**. (**b**) Corresponding normalized autocorrelation function.

Dynamic light scattering (DLS) and Z-potential (Z-pot) measurements: DLS and Z-pot were used to study the colloidal dispersions of **Er_2_-W_34_** (5 mM in distilled water). First, the hydrodynamic diameter (*D*_h_) of **Er_2_-W_34_** was determined both at pH 5.6, the pH at which the NMR relaxivities of POMs were studied, and at pH 7 to investigate their stability at physiologically relevant pH values (see Supplementary Information for details). As shown in Figure 5, at pH 5.6, the POMs have an average *D*_h_ of 1.2 ± 0.4 nm with a polydispersity index (PDI) of 0.147, indicating good colloidal stability in water. A comparable colloidal stability was observed at pH 7.0 (Figure 6) with a very similar hydrodynamic size (*D*_h_ = 1.3 ± 0.4 nm; PDI = 0.193). However, a second population of particles with a larger mean *D*_h_ of 176.6 ± 71.1 nm was also observed. These larger particle aggregates can most likely be explained as sodium hydroxide solution was used to adjust the pH of the particle dispersion, and the presence of positively charged Na^+^ ions could promote aggregation between particles through electrostatic interactions [42]. The net negative charge of **Er_2_-W_34_** when dispersed in water was confirmed by Z-pot measurements. At pH 5.6, the POMs have a negative Z-pot of −9.0 ± 0.8 mV, while at neutral pH the Z-pot increased towards more negative values of −20.4 ± 0.1 mV. The more negative Z-potential value at neutral pH for **Er_2_-W_34_** can be explained by the presence of more deprotonated oxygen groups at more basic pH, which contribute to a larger number of negative charges.

**Figure 7:**
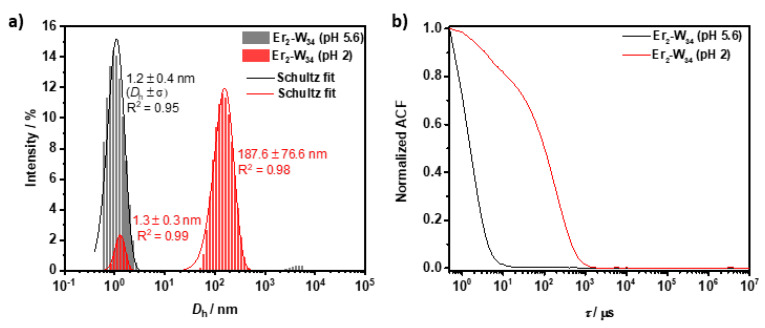
DLS analysis on **Er_2_-W_34_** (5 mM) at different pH values of the particle dispersion. (**a**) Hydrodynamic diameter of **Er_2_-W_34_** at pH 5.6 (gray) and at 40 min after addition of HCl_aq_ (37%, 3 µL to 500 µL of sample) at pH 2. (**b**) Corresponding normalized autocorrelation function.

As the anionic units of **Er_2_-W_34_** are linked by two acetate groups, the possibility of a change in the aggregation state of the POM upon addition of acid was further investigated by DLS analysis. The acetate linker (p*K*_a,acetic acid_ = 4.7) is expected to be completely protonated in strongly acidic media (pH 2) and loses its coordination ability, leading to a significant change in the aggregation state of the POM. To test this hypothesis, the hydrodynamic diameter was measured before (pH 5.6) and 40 min after acid addition (HCl_aq_, pH 2). As shown in Figure 7, it can be observed that at pH 2 the scattering intensity of the initial population of small particles (1.2 nm) decreases greatly, while a second population of particles with a *D*_h_ of about 187 nm is formed. The formation of this second and larger population of particles can most likely be attributed to the degradation of the small particles leading to the formation of large aggregates. This data suggests that, unless the pH of the particle dispersion is acidified (pH < 4.7), the dimeric structure of **Er_2_-W_34_** is preserved in water.

Fourier transform infrared (FTIR) spectroscopy: FTIR spectroscopy was carried out on dried **Er_2_-W_34_** sample, that was obtained by evaporating the aqueous solvents. FTIR spectra of the recovered **Er_2_-W_34_** show the expected absorption bands ascribed to the pristine POM. The two spectra perfectly match each other and no wavenumber shifts are observed indicating the stability of the **Er_2_-W_34_** in solution state (Figure S1).

Xylenol Orange Test: In addition to the instrumental analysis to check the integrity of **Er_2_-W_34_** in solution, a quick visual Xylenol Orange test was performed to detect the presence of free Er(III) ions. Xylenol Orange test allows an assessment of the amounts of free metal ions and free ligand in a solution of a given lanthanide containing complex. In the presence of free lanthanide ions, the xylenol orange solution undergoes a color change from orange to violet that can be visually detected [43]. No color change was observed on addition of Xylenol Orange (30 µL) to the **Er_2_-W_34_** solution (pH 5.6) which was used for NMR relaxivity studies. This indicate that the Er(III) ions do not leach out into solution. Whereas, violet color was observed upon addition of Xylenol Orange to the **Er_2_-W_34_** solution containing ca. 2 mg Er(NO_3_)_3_·6H_2_O as a source of free Er(III) ions (Figure S3).

Based on the above conducted experiments (DLS, post FTIR and Xylenol Orange tests) the structural integrity of **Er_2_-W_34_** in solution can be confirmed.

### 2.4. Longitudinal Relaxivity r_1_

**Figure 8:**
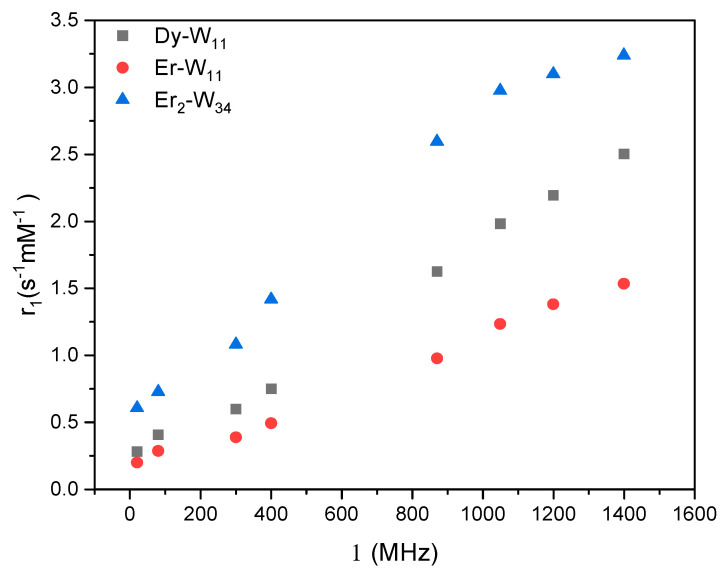
PRE as measured by longitudinal relaxivities *r*_1_ of ^1^H of water with POM metal clusters (**Dy-W_11_** and **Er-W_11_**, and **Er_2_-W_34_**) as a function of the Larmor frequency ν. In first approximation, the PRE in the presence of the paramagnetic metal clusters increases linearly with the applied magnetic field except for **Er_2_-W_34_**, where a levelling-off is observed at high Larmor frequencies.

The longitudinal relaxation rates *R*_1_(*c*) of the studied POM metal complexes show a linear dependence with concentration *c,* which leads to the calculation of the slope (i.e., the longitudinal relaxivity *r*_1_). Please note that the concentration used for the relaxivity is the one of the entire POM metal complex rather than the Ln-ion. For all compounds *r*_1_ monotonically increases with Larmor frequency (i.e., with magnetic field (Figure 8)). Among these three POM clusters (**Er_2_-W_34_**, **Dy-W_11_** and **Er-W_11_**) **Er_2_-W_34_** shows the highest longitudinal relaxivity.

Taking the data from Table 1, the magnetic moment of Dy^III^ (10.6 μ_B_) is higher than the one of Er^III^ (9.6 μ_B_). Thus, the higher longitudinal relaxivity of **Dy-W_11_** compared with **Er-W_11_** supports the influence of electron spin of the paramagnetic centers on PRE, since, in these POMs, single paramagnetic metal centers (Dy^III^ and Er^III^) are incorporated within similar diamagnetic Keggin moieties. As already mentioned, another important factor that determines the inner sphere contribution to longitudinal relaxivity is the number of exchangeable water molecules that are directly associated with the paramagnetic centers of the complexes. Assuming fast exchange, the longitudinal relaxivity is directly proportional to the hydration number *q* and the residence time of the water molecules. In the case of **Dy-W_11_** and **Er-W_11_** POMs, Dy^III^ and Er^III^ ions have four and three exchangeable aqua ligands, respectively. The difference in the number of water ^1^H in {Dy(H_2_O)_4_}^3+^ and {Er(H_2_O)_3_}^3+^ also contributes to the higher longitudinal relaxivity of **Dy-W_11_** compared with that of **Er-W_11_**. Other relevant properties that can account for the difference in relaxivities of **Dy-W_11_** and **Er-W_11_** are the ionic radii of Dy^III^ and Er^III^, their magnetic anisotropies and the electron spin relaxation times [12].

The relaxivities of **Er_2_-W_34_** are more than doubled compared to **Er-W_11_** and this ratio is enhanced towards lower frequencies. Moreover, *r*_1_ of **Er_2_-W_34_** levels off a very high field. In order to explain these differences in relaxivity dispersion, several factors need to be considered. First, the magnetic moment of dimerized Wells-Dawson POM **Er_2_-W_34_** is twice the magnetic moment of **Er-W_11_**. This can cause increase of the relaxivitivy. However, for a detailed quantitative modelling further information is needed on the correlations and couplings between the two Er^III^ ions in the dimer. Second, in the chemical structure of the **Er_2_-W_34_**, only one of the eight coordination sites of each paramagnetic Er^III^ is coordinated by a single water molecule in the solid-state, which limits the possibility of chemical exchange, and hence reduces the PRE. Third, the Wells-Dawson POM **Er_2_-W_3_**_4_ (2.7 × 0.9 nm in size) has a bigger molecular weight compared to **Er-W_11_** (1.1 × 1.0 nm in size). This causes lower tumbling rates and different diffusion behavior that can explain the relative enhancement of *r*_1_ of **Er_2_-W_34_** with respect to **Er-W_11_** at low fields and the levelling-off at highest fields. However, with the available set of data, our model is rather qualitative at the current state, but makes the observed PRE behavior plausible. More systematic studies of other Ln_2_-W_34_ complexes are needed to identify and quantify the microscopic mecanisms that are responsible for the observed PRE behavior in these highly complex systems.

### 2.5. Transverse Relaxivity r_2_

**Figure 9:**
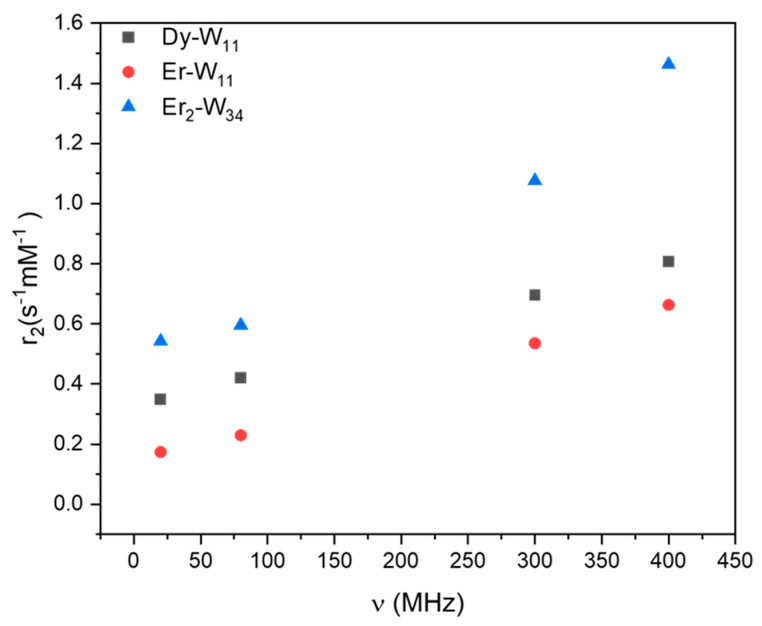
PRE measured by transverse relaxivities *r*_2_ of ^1^H of water with POM metal clusters as a function of the Larmor frequency ν. *r*_2_ of **Dy-W_11_** and **Er-W_11_** show an approximately linear dependence with the applied magnetic field for Larmor frequencies up to 400 MHz. For **Er_2_-W_34_** a deviation occurs above 300 MHz, probably due to a Curie-contribution due to the large size of the molecule.

For the application of a material as an MRI contrasting agent, the significance of transverse relaxivity *r*_2_, which causes negative image contrasts, is as important as the longitudinal relaxivity *r*_1_. In the present studies, transverse relaxivities of the POMs also show approximately linear field dependence due to the PRE effect (Figure 9). The higher relaxivity *r*_2_ of **Dy-W_11_** compared to **Er-W_11_** can be correlated with the effective magnetic moment of the paramagnetic center since *r*_2_ is proportional to the square of the magnetic moment of the lanthanide ions. Dy^III^ has a higher magnetic moment of 10.6 µ_B_ than Er^III^ which has *µ*_eff_ = 9.6 µ_B_. The comparison of **Er_2_-W_34_** and **Er-W_11_** in (Figure 9) indicates that the PRE induced transverse relaxivity *r*_2_ of the POM metal complexes also depends on the number of paramagnetic centers in the molecule. Moreover, the *r*_2_ of **Er_2_-W_34_** starts to deviate from a linear field dependence above 300 MHz. This behavior is consistent with an increasing Curie-spin contribution to the relaxivity. This term has a quadratic field dependence and becomes enhanced for large molecules and at high magnetic field [41,44].

Measurements of *r*_2_ in a very high magnetic field (≥800 MHz) were not carried out on the investigated samples due to the large *T*_2_ values of the complexes compared to the field fluctuations of the resistive magnet.

## 3. Experimental Section

### 3.1. ^1^H—Frequencies 870–1400 MHz

High field NMR relaxivity studies were accomplished at the Laboratoire National des Champs Magnétiques Intense (LNCMI) in Grenoble equipped with 24 MW resistive magnet providing variable fields up to 35 T in a 34 mm room temperature bore. To overcome the low field homogeneity (50 ppm/mm at 1 mm off-center position) of these magnets, small sample volumes (1 mm^3^) were used that were precisely centered (better than 0.2 mm) at the center of the magnet. For this purpose, a specially designed sample filling system was developed to ensure the placement of precise sample volumes at the center of capillary tubes (10 mm length and 1 mm inner diameter). Moreover, single scan inversion recovery measurements were used to reduce the impact of fast field fluctuations (up to 50 ppm) on the measurement. For the experiment, a specially designed single-resonance ^1^H-NMR probe was used enabling in situ tuning of NMR frequencies between 850 MHz and 1.4 GHz. Data acquisition and data analysis were performed by using a home-built variable-frequency NMR spectrometer covering Larmor frequency up to 2 GHz and specially designed data acquisition software. As resistive magnets provide access to variable fields (field ramp rate of 5 T/min), the relaxation rates for each concentration were measured at all the frequencies 870 MHz (20.4 T), 1050 MHz (24.7 T), 1200 MHz (28.2 T), and 1400 MHz (33 T) using the same magnet.

### 3.2. ^1^H—Frequencies 20–400 MHz

The relaxation measurements at lower magnetic fields were measured on commercially available NMR instruments. The experimental details are described in [20,21] to which we refer here.

## 4. Conclusions

Water ^1^H relaxivities are a determining factor for assessing the effectiveness of MRI contrast agents. Often, paramagnetic systems with higher relaxivities result in images with better contrast. PRE of water has been investigated over a large range of magnetic fields for aqueous solutions for the paramagnetic POM clusters **Dy-W_11_**, **Er-W_11_** and **Er_2_-W_34_**. The *r*_1_ and *r*_2_ values are the fitted slopes of *R*_1_ and *R*_2_ as a function of POM concentration, respectively. As a basis for interpretation, we compare the PRE of the three compounds. POMs with paramagnetic Dy^III^ and Er^III^ ions have very short electronic relaxation times compared to their Gd^III^ analogs and are thus efficient relaxing agents at intermediate and high magnetic fields. The short electronic relaxation time is due to the highly anisotropic electronic ground state of these ions. In addition to the total number of unpaired electrons, magnetic moment and the anisotropic nature of paramagnetic ions prove to be key for the development of efficient CAs for high field MR imaging. There is a need to track these effects through systematic experimental studies on the relaxation properties of paramagnetic compounds.

## Figures and Tables

**Table 1:** Magnetic properties of non-interacting lanthanide ions.

Ln(III)	Dy(III)	Er(III)
Free-ion ground state term ^2S+1^L_J_	^6^H_15/2_	^4^I_15/2_
*S*	5/2	3/2
*L*	5	6
*J*	15/2	15/2
*g*-factor	4/3	6/5
$µeff=gJµB\sqrt{J\left(J+1\right)}$	10.6	9.6
χT expected value for non-interacting ions per complex (cm^3^Kmol^−1^)	14.7	11.5

## Data Availability

Not applicable.

## Supplementary Materials

The following are available online, Figure S1: Comparison of FTIR spectra of Er2-W34, Figure S2: Pictures of NMR tubes containing Er2-W34 solution after NMR relaxivity studies, Figure S3: Pictures of NMR tubes: Upon addition of xylenol orange solution to the Er2-W34 solution after NMR relaxivity studies. Table S1. Magnetic properties of isolated lanthanide ions

## Sample Availability

Samples of the compounds K_5_[Dy(H_2_O)_4_GeW_11_O_39_]·16H_2_O (**Dy-W_11_**), K_5_[Er(H_2_O)_3_GeW_11_O_39_]·20H_2_O (**Er-W_11_**), and (NH_2_Me_2_)_13_Na_3_[{Er(H_2_O)(CH_3_COO)(P_2_W_17_O_61_)}_2_]·40H_2_O (**Er_2_-W_34_**) [{Er^III^(H_2_O)(CH_3_COO)(P_2_W_17_O_61_)}_2_]^16−^ (**Er_2_-W_34_**) are available from the authors.
