# Supplementary material for: NMR Relaxivities of Paramagnetic Lanthanide-Containing Polyoxometalates
Source: Molecules. 2021 Dec 10;26(24):7481. doi: 10.3390/molecules26247481 (PMC8703889; doi:10.3390/molecules26247481)
Supplement: Supplementary file 1 [file molecules-26-07481-s001.zip › molecules-1438892-supplementary.pdf]

Electronic Supplementary Information (ESI)

NMR Relaxivities of Paramagnetic Lanthanide-Containing Polyoxometalates

Aiswarya Chalikunnath Venu[1], Rami Nasser Din[2], Thomas Rudszuck[3], Pierre Picchetti[1], Papri Chakraborty[1], Annie K. Powell[1,4,5]*, Steffen Krämer[2]*, Gisela Guthausen[3,6]*, Masooma Ibrahim[1]*

Experimental

Materials and methods

Synthesis: The lanthanide-containing polyoxometalates (POMs), $K_5[Dy(H_2O)_4GeW_{11}O_{39}]\cdot16H_2O$ (Dy-W_{11}), $K_5[Er(H_2O)_3GeW_{11}O_{39}]\cdot20H_2O$ (Er-W_{11}) [1], and $(NH_2Me_2)_{13}Na_3[\{Er(H_2O)(CH_3COO)(P_2W_{17}O_{61})\}_2]\cdot40$ H_2O **(Er_2-W_{34})** $[\{Er^{III}(H_2O)(CH_3COO)(P_2W_{17}O_{61})\}_2]^{16-}$ **(Er_2-W_{34})** [2] were synthesized according to the literature methods and was characterized by FTIR spectroscopy. Xylenol Orange solution was prepare in 0.5 M LiOAc/HOAc buffer (pH 5.6) with slight modification from literature method [3]. All reactions were carried out under aerobic conditions. All other reagents were commercially purchased and were used without further purification.

Electrospray ionization mass spectrometry (ESI MS): The solution and gas phase behavior of **Er_2-W_{34}** was studied using electrospray ionization mass spectrometry (ESI MS). A Waters Synapt G2S system was used for this purpose and the samples were measured in the negative ion mode. 0.2 mM solution from NMR tube were further diluted with acetonitrile (ACN) and H_2O at the 50:50 (v/v) ratio.

Dynamic Light Scattering (DLS) Measurements: DLS measurements were performed by using a Malvern ZetaSizer nano device. Prior to measurement, samples were filtered through a syringe filter (0.22 µm, polypropylene membrane). The intensity of the scattered light was measured at a fixed angle (173°), while the laser light wavelength for the scattering experiments was λ=633 nm. The refractive index for POMs was 1.55. Data analysis was performed according to standard procedures by using the Malvern software. Briefly, the decay rates were determined by the following relationship, where η is the viscosity of the medium (water), D_h is the hydrodynamic diameter, and θ is the scattering angle.

$$\Gamma = D_h \cdot \left(\frac{4\pi\eta}{\lambda} \cdot \sin\left(\frac{\theta}{2}\right)\right)^2$$

To fit the autocorrelation function, the non-negatively constrained least squares (NNLS) algorithm built into the software was used, which allows for the determination of the diffusion coefficient (d), from which the hydrodynamic diameter of the particles is calculated by using the Stokes-Einstein equation (see below), where k_b is the Boltzmann constant, T is the temperature, and η is the viscosity of the medium (water).

$$D_h = \frac{k_b \cdot T}{3 \cdot \pi \cdot \eta \cdot d}$$

Zeta-potential measurements were performed by using the Malvern ZetaSizer nano device and analyzed according to standard procedures using Malvern software.

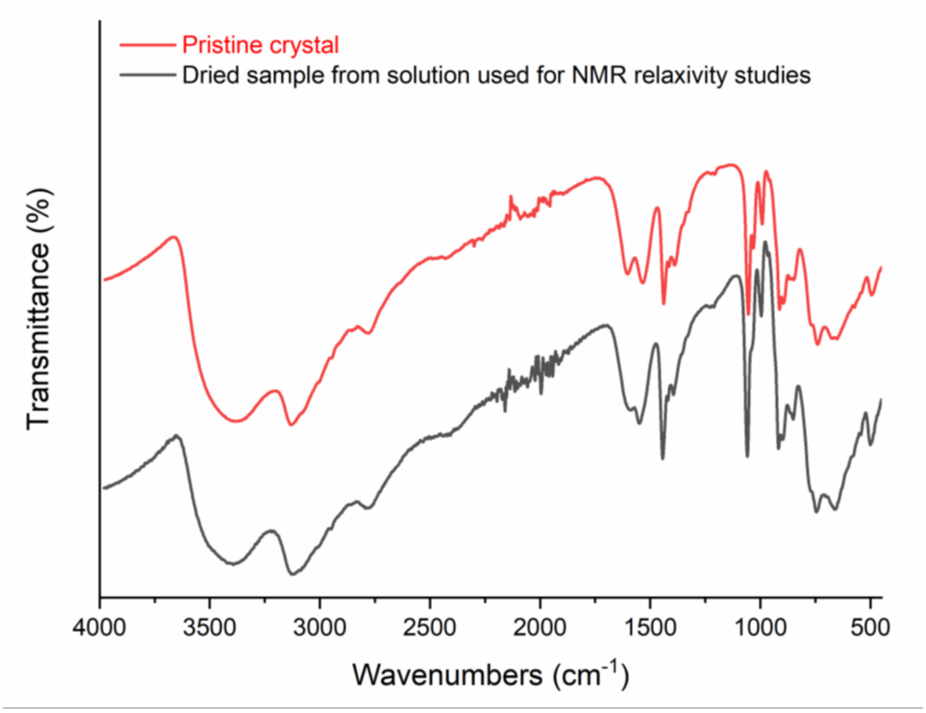

Figure S1. Comparison of FTIR spectra of **Er_2-W_{34}:** Pristine crystals (red) and dried sample obtained from solution used for NMR relaxivity measurement (black).

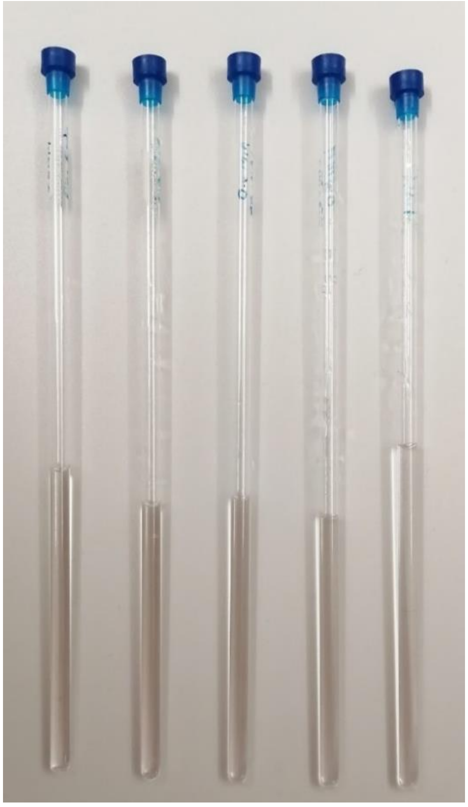

Figure S2. Pictures of NMR tubes containing **Er_2-W_{34}** solution after NMR relaxivity studies.

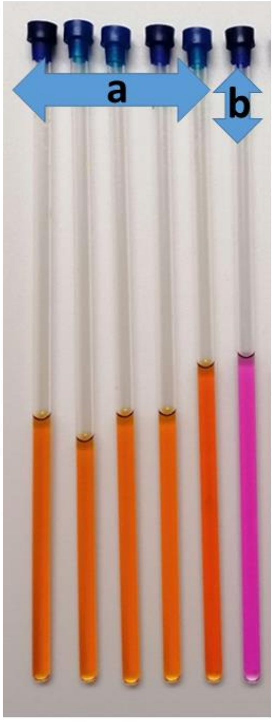

Figure S3. Pictures of NMR tubes: Upon addition of xylenol orange solution to the **Er_2-W_{34}** solution after NMR relaxivity studies (a) Upon addition of xylenol orange solution to the **Er_2-W_{34}** solution containing ca. 0.2 mg of $Er(NO_3)_3 \cdot 6H_2O$.

Table S1. Magnetic properties of isolated lanthanide ions

Ln^{III}	$4f^n$ electron configuration	Free-ion ground state term $^{2S+1}L_J$	Free-ion g value	$\mu eff = gJ\mu B\sqrt{J(J+1)}$	8-coordinate radius	4f electron density distribution's geometry	Free-ion $\chi_m T_{calc}$ value at RT ($cm^3\ K\ mol^{-1}$)
**La**	$4f^0$	$^1S_0$	------	------	1.16	---------	0
**Ce**	$4f^1$	$^2F_{5/2}$	6/7	2.54	1.14	Oblate	0.80
**Pr**	$4f^2$	$^3H_4$	4/5	3.58	1.13	Oblate	1.60
**Nd**	$4f^3$	$^4I_{9/2}$	8/11	3.68	1.11	Oblate	1.64
**Pm**	$4f^4$	$^5I_4$	3/5	2.68	1.09	Prolate	0.90
**Sm**	$4f^5$	$^6H_{5/2}$	2/7	0.85	1.08	Prolate	0.09
**Eu**	$4f^6$	$^7F_0$	-----	-----	1.07	Isotropic	0
**Gd**	$4f^7$	$^8S_{7/2}$	2	7.94	1.05	Isotropic	7.88
**Tb**	$4f^8$	$^7F_6$	3/2	9.7	1.04	Oblate	11.82
**Dy**	$4f^9$	$^6H_{15/2}$	4/3	10.6	1.03	Oblate	14.17
**Ho**	$4f^{10}$	$^5H_8$	5/4	10.6	1.02	Oblate	14.07
**Er**	$4f^{11}$	$^4I_{15/2}$	6/5	9.6	1.00	Prolate	11.48
**Tm**	$4f^{12}$	$^3H_6$	7/6	7.6	0.99	Prolate	7.15
**Yb**	$4f^{13}$	$^2F_{7/2}$	8/7	4.5	0.99	Prolate	2.57
**Lu**	$4f^{14}$	$^1S_0$	-----	-----	0.98	Isotropic	0

$g_J = 3/2 + [S(S+1)-L(L+1)]/2J(J+1)$ (Landé factor, g_J is the g-factor for a spin and orbital factors taken together).
$\chi_m T_{calc} = g^2/8*J(J+1)$.
RT = room temperature.
χ_m = molar magnetic susceptibility
τ_e = relaxation time
μB = Bohr magneton
μ_{eff} = effective Bohr magneton number

References

1. Ibrahim, M.; Mbomekallé, I.M.; de Oliveira, P.; Baksi, A.; Carter, A.B.; Peng, Y.; Bergfeldt, T.; Malik, S.; Anson, C.E. Syntheses, crystal structure, electrocatalytic, and magnetic properties of the monolanthanide-containing germanotungstates $[Ln(H_2O)_nGeW_{11}O_{39}]^{5-}$ (Ln = Dy, Er, n = 4,3). *ACS Omega.* **2019**, *4*, 21873–21882.
2. Ibrahim, M.; Baksi, A.; Peng, Y.; Al-zeidaneen, F.K.; Mbomekall, I.M.; Oliveira, P. De; Anson, C.E. Synthesis, characterization, electrochemistry, photoluminescence and magnetic properties of a dinuclear erbium(III)-containing monolacunary Dawson-type tungstophosphate: $[\{Er(H_2O)(CH_3COO)(P_2W_{17}O_{61})\}_2]^{16-}$. *Molecules*. **2020**, *25*, 4229.
3. Barge, A.; Cravotto, G.; Gianolio, E.; Fedeli, F. How to determine free Gd and free ligand in solution of Gd chelates. A technical note. *Contrast Media Mol. Imaging* **2006**, *1*, 184–188.
